# Determine the iodine content of salt at the household level and its predictors in Bahirdar Town, Northwest Ethiopia

**DOI:** 10.11604/pamj.2022.41.260.17910

**Published:** 2022-03-30

**Authors:** Temesgen Mersha, Terefe Derso, Musa Jemal, Shemsu Kedir, Bekri Mohammed

**Affiliations:** 1 Department of Human Nutrition, Institute of Public Health, College of Medicine and Health Sciences, University of Gondar, Gondar, Ethiopia,; 2 Department of Public Health, College of Medicine and Health Science, Werabe University, Werabe, Ethiopia

**Keywords:** Salt iodization, iodine deficiency, households, Bahirdar, Ethiopia

## Abstract

**Introduction:**

iodine deficiency remains a foremost public health problem in developing countries. About 66 million populations were at risk from iodine deficiency, 28 million people suffer from goiter and more than 50,000 prenatal deaths are related to iodine deficiency each year in Ethiopia. Besides, studies from different parts of Ethiopia have shown that a low proportion of households use adequate iodine concentration and varied from one household to another. Despite increased coverage, the quality of available salt is poor. To ensure safe and effective levels of iodine consumption, monitoring the levels of iodine in salt and the iodine status of the population is critical. However, kinds of literature are scant in Ethiopia particularly; no study is conducted in the current study area. Thus, the study aimed to determine the iodine content of salt and associated factors at the household level in Bahir Dar Town, Northwest Ethiopia.

**Methods:**

a community based cross-sectional study design was carried out in Bahir Dar Town from September to October 2015. A multi-stage sampling technique was used to select 706 study participants. A pre-tested, structured questionnaire and laboratory were used to collect data. A laboratory test, gold standard iodometric titration method was used to measure individual availability of adequately iodized salt. Multivariable logistic regression analysis was fitted to identify factors associated with the content of iodine. Adjusted Odds Ratio (AOR) with corresponding 95% confidence interval was computed to show the strength of association. In multivariable analysis, a p-value of <0.05 was used to declare statistical significance.

**Results:**

a total, of 690 participants were included in the study. About 70.1% (95%CI: 63.41, 76.76) of the households were used adequate iodized salt (≥15 ppm). The result of the multivariate analysis revealed that respondents with secondary school (AOR=3.05; 95%CI: 1.51,6.18), age 30-44 years (AOR=1.99; 95%CI: 1.08,3.69), good knowledge (AOR=3.34; 95% CI: 2.09,5.32) and being in the highest wealth status (AOR=4.35,95% CI: 2.43,7.8) had higher odds of availability of adequately iodized salt at the household compared to the counterpart. Besides, using covered salt (AOR=6.10, 95% CI: 3.78, 9.87) and storing salt in a dry place (AOR=4.17; 95% CI: 2.21, 7.86) were positively associated with the availability of adequately iodized salt.

**Conclusion:**

the availability of adequately iodized salt in the household is still low. Further institutionalizing iodized salt regulation and awareness creation will require to improve safe iodine consumption through the community.

## Introduction

More than two billion people, 28% in the world and most of them in developing countries (39% in Africa) suffer from inadequate intake of iodine [1,2]. Iodine deficiency causes a wide range of health-related problems that are collectively called iodine deficiency disorders (IDD) characterized by mental impairment, goiter, hypothyroidism, and dwarfism [1-3]. Groups at the highest risk to suffer from iodine deficiency are lactating women, pregnant women, and children younger than three years [1,2]. The iodization of salt, a common food that is used by a majority of the population, is a confirmed intervention for the prevention of IDD [4] and in Africa, saved millions of children from its adverse effects, mainly due to the increased household availability of iodized salt [5]. Iodine is an essential trace mineral required in small amounts for the normal physiologic function of humans which is necessary for regulating metabolic rate, growth, and development of body structures, and neuronal function and development [6,7].

Nonetheless, different studies revealed that the availability of adequate iodized salt was low in the households: in sub-Saharan Africa 59%, South Asia 69%, Somalia 4% and Mauritania 7% [8], India 42% [9], Vietnam 73.6% [10], Kenya 26.2% [11], Pakistan 15% [12] and Sudan 14.4% [13]. According to different studies, factors that are associated with the availability of adequately iodized salt at the household includes: using packed salt [14,15], not exposing salt to sunlight [14,15], storage of salt in dry areas [11], shorter storage of salt [14] and add salt late at the end of cooking [16]. Besides, socio-demographic factors (formal education) [14,17] and high socioeconomic status (income) [18] and good knowledge about iodized salt and IDD [14,17] were significantly associated with the availability of adequately iodized salt.

About 66 million populations were at risk from iodine deficiency [19], 28 million people suffer from goiter and more than 50,000 prenatal deaths are related to iodine deficiency each year in Ethiopia [20]. Besides, studies from different parts of Ethiopia have shown that a low proportion of households uses adequate iodine concentration i.e. below 30%, and iodine concentration varied from one household to another [14,17,20,21]. Despite increased coverage of iodized salt, the quality of available salt is poor (16). To ensure safe and effective levels of iodine consumption, monitoring the levels of iodine in salt and the iodine status of the population is critical [22,23]. However, a few studies in Ethiopia were conducted to determine the amount of iodine content in the salt at the household level by using qualitative kits, which have low specificity, resulting in high numbers of false positives, and particularly; no study is conducted in the current study area. Thus, the study aimed to determine the iodine content of salt and associated factors at the household level in Bahir Dar Town, Northwest Ethiopia by using the gold standard iodometric titration method.

## Methods

**Study area and study period:** a community based cross-sectional study design was carried out in Bahir Dar Town from September to October 2015. The town is the capital city of the Amhara region state, located 568 km away from Northwest of Addis Ababa, the capital of Ethiopia. A total, of 311,725 populations resides in the town [24]. The town has nine sub-city administrations and there are five iodized salt whole distributors and one producer (repacker) in the town.

**Study participants:** all households in selected sub-cities of Bahirdar Town were included in the study.

**Sample size and sampling procedure:** the sample size was calculated using Epi-info version 7 by considering the following assumptions; 28.9% prevalence of expected households using adequately iodized salt [14], 95% level of confidence, 5% margin of error, 5% non-response rate, and a design effect of 2. Thus, a minimum sample size of 706 was obtained. Regarding the sampling procedure, multi-stage sampling techniques were used to select the study subjects. Firstly, three sub-cities (Tana, Shimbit, and Gishabay sub-city) were randomly selected out of nine sub-cities in the town. Accordingly, a total number of households was proportionally allocated for each sub-city (1 to 3). In the end, households from each sub-city were selected using a systematic sampling method.

**Data collection tools and procedures:** the pretested, structured, and interviewer-administered questionnaire and laboratory test were used to collect data. The questionnaire was composed of socio-demographic, personal factors (knowledge about iodized salt and IDD), and environmental factors (salt storage place, duration of storage, type of packaging, moisture, heat/sunlight, source of salt). Initially, the English version questionnaire was translated into Amharic, the native language of the study area, and then back-translated to English, to maintain its consistency. The questionnaire was pretested out of the study area. During the pre-test, the acceptability and applicability of procedures and tools were evaluated, six and two were recruited as data collectors and supervisors, respectively. Intensive training was given to data collectors and supervisors for two days. The daily check-up was made on the completed questionnaire during submission.

The household wealth index was computed using composite indicators for urban residents considering the following assets; ownership of the house, selected household assets, and bicycle, motorcycle, and automobile. Principal component analysis (PCA) was performed to determine the households´ wealth index, and then the wealth status of the study participants was categorized into 3: poor, medium, and highest.

**Knowledge:** respondents who scored 50% and above regarding iodized salt knowledge questions were leveled as good knowledge, whereas below 50% scored leveled as poor knowledge [14].

**Measurement of iodine in the salt:** in a laboratory test, the gold standard iodometric titration method was used to measure individual availability of adequate iodized salt [25]. From each household, approximately 50g and homogenized salt samples were collected and transported to the laboratory daily using a moisture-free, clean, and airtight plastic container. The sample was labeled and coded with the following information during collection: date of sampling, source of salt, batch number, production date, and expiry date. The iodometric titration involved the use of reagents: sulphuric acid, potassium iodate, and potassium iodide as principal reagents, standardized sodium thiosulphates (as titrant), and starch solution (as an indicator). A trained analyst using the gold standard iodometric titration method did the laboratory test. Each sample was analyzed in triplicate and the average of these was taken as the iodine concentration of the sample. The conversion of the titration results to iodine concentrations was done using a standardized table as per recommendations of the UNICEF/WHO [26]. Adequately iodized salt at the household level was defined as a salt sample that has ≥15 parts per million (PPM) of iodine, whereas below <15ppm is defined as inadequately iodized salt.

**Data management and analysis:** data were entered into Epi-info version 3.5.3 and exported to SPSS version 20 for analysis. Descriptive statistics using frequencies and proportions were used to summarize the study variables. A binary logistic regression model was fitted. Both Crude Odds Ratio (COR) and Adjusted Odds Ratio (AOR) with corresponding 95% Confidence Interval (CI) were computed to show the strength of association. In multivariable analysis, variables with P-value<0.05 were considered statistically significant.

**Ethical considerations:** ethical clearance was obtained from the institutional review boards of the University of Gondar. Permission was obtained from Amhara Regional State Health Bureau, Bahir Dar Town Zone, Health Department, and the selected sub-cities. In addition, the purpose and the importance of the study were explained and verbal informed consent was secured from each household. Respondents were also informed that they could refuse or discontinue participation at any time. Information was recorded anonymously to maintain the confidentiality and privacy of the respondent.

## Results

**Socio-demographic characteristics of the study participants:** a total of 690 household respondents were included in the study with a response rate of 97.73%. The mean (SD) age of the respondent was 34.5 years (SD ± 11.77). Besides, 29.1% and 33.2% of respondents were secondary education and housewife, respectively (Table 1).

**Table 1:** sociodemographic characteristics of study participants in Bahir Dar Town, Northwest Ethiopia, 2015

Characteristics (N=690)	Category	Frequency (N)	Percent (%)
Age of the respondent	18-29 years	307	44.5
	30-44 years	238	34.5
	≥45 years	145	21.0
Sex of the respondent	Female	635	92.0
	Male	55	8.0
Marital status of the HH	Married	429	62.2
	Single	125	18.1
	Divorced	52	7.5
	Separate	45	6.5
	Widowed	39	5.7
Educational status of respondents	Unable to read and write	80	11.6
	Able read and write	87	12.6
	Primary school	129	18.7
	Secondary school	201	29.1
	Above secondary school	193	28.0
Occupation of respondents	Housewife	214	33.23
	Government employee	181	28.11
	Merchant	146	22.67
	NGO/private work	53	8.23
	Daily laborer	50	7.76
Family size	<5	575	83.3
	>5	115	16.7
Wealth index	Poor	223	32.3
	Medium	239	34.6
	Rich	228	33.1

HH: household; NGO: non-governmental organization

**Knowledge and practice of respondents regarding iodized salt:** the vast majority (95.36%) and 86.1% of the respondents used powdered and packed iodized salt, and were stored in salt and in a dry place away from humidity and heat/fire area, respectively. Besides, more than three-fourths (80.6%) of the respondents had good knowledge about iodized salt and IDD (Table 2).

**Table 2:** knowledge and practice of respondents regarding iodized salt in Bahir Dar Town, North West Ethiopia, 2015/16

Variables	Category	Frequency (N)	Percent (%)
Knowledge of the respondent	Good knowledge	556	80.6
	Poor knowledge	134	19.4
Type of salt used	Powdered and packed salt	658	95.36
	Crystalline	32	4.64
Source of salt	Village shop	468	67.67
	Supermarket	123	17.97
	Market day	99	14.35
Use cover for the salt container	Yes	568	82.32
	No	122	17.68
Salt storage place	Dry area	563	81.59
	Moisture	71	10.29
	Heat/fire	56	8.12
Duration of salt storage	Short	508	73.6
	Long	182	26.4
Washing salt	No	692	99.28
	Yes	5	0.72

**Figure 1:**
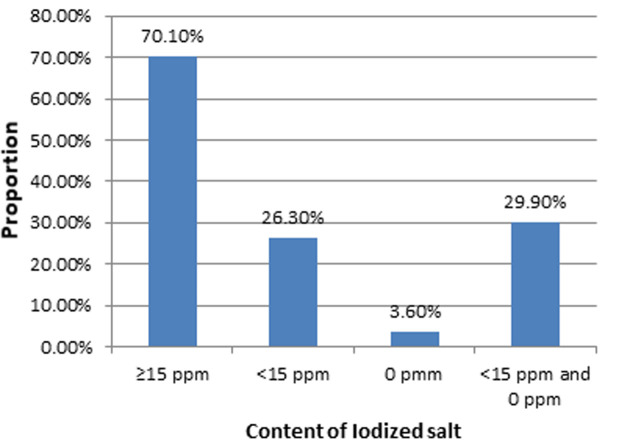
iodine content of salt at household level in Bahir Dar Town, Northwest, Ethiopia, 2015

**Availability of adequately iodized salt at the household level:** about 70.1% (95%CI: 63.41, 76.76) of the households had adequately iodized salt (≥15 ppm) (Figure 1).

**Factors associated with the availability of iodized salt at household level:** the result of the multivariate analysis revealed that respondents with secondary school (AOR=3.05; 95%CI: 1.51,6.18), age 30-44 years (AOR=1.99; 95%CI: 1.08,3.69), good knowledge (AOR=3.34; 95%CI: 2.09,5.32) and being in the highest wealth status (AOR=4.35,95% CI: 2.43,7.8) had higher odds of availability of adequately iodized salt at the household. Besides, using covered salt (AOR=6.10, 95% CI: 3.78, 9.87) and storing salt in dry places (AOR=4.17; 95% CI: 2.21, 7.86) were positively associated with the availability of adequately iodized salt (Table 3).

**Table 3:** factors associated with the availability of iodized salt at household level in Bahir Dar Town, Northwest, Ethiopia, 2015

Variables	Iodine content		COR (95% CI)	AOR (95% CI)
	≥15 ppm	<15 ppm		
**Age of respondent**				
18-29 years	205	102	1.16 (0.77,1.75)*	1.27 (0.68,2.38)
30-44 years	187	51	2.11 (1.34,3.34)	1.99 (1.08,3.69)
≥45 years	92	53	1	1
**Educational status of the respondents**				
Unable to read and write	45	35	1	1
Able read and write	53	34	2.00 (1.08,3.71)	1.17 (0.56,2.42)
Primary school	87	42	2.66 (1.49,4.73)	1.88 (0.96,3.67)
Secondary school	163	38	5.52 (3.13,9.71)	6.35 (3.15,12.8)
Above secondary school	146	47	3.99 (2.30,6.93)	3.05 (1.51,6.18)
**Use covered salt**				
Yes	435	133	4.87 (3.23,7.35)	6.10 (3.78,9.87)
No	49	73	1	1
**Salt storage place**				
Dry area	425	138	3.55 (2.03,6.22)	4.17 (2.21,7.86)
Moisture area	33	38	1.00 (0.49,2.02)	1.30 (0.58,2.96)
Near heat/fire area	26	30	1	1
**Knowledge of the respondent**				
Good knowledge	422	134	3.66 (2.47,5.41)	3.34 (2.09,5.32)
Poor knowledge	62	72	1	1
**Wealth index**				
Poor	131	92	1	1
Medium	176	63	1.96 (1.33,2.90)	1.79 (1.09,2.96)
Highest	177	51	2.44 (1.62,3.67)	4.35 (2.43,7.8)

## Discussion

The current study revealed that about 70.1% (95%CI: 63.41, 76.76) of the households had adequately iodized salt (≥15 ppm). The findings in South Asia 69% [8] and Vietnam 73.6% [10] have the same result. On the other hand, the present finding was higher than compared to the other findings: in Ethiopia (Gondar Town 28.9% [14], Laelay Maychew District 33% [17] and Assosa Town 26.1% [27]), Somalia 4% and Mauritania 7% [8], Malaysia 6.8% [28], Pakistan (15%) [12] and India 42% [9]. The possible reason for the higher proportion of iodine content in the households in the present study may be that during the past five years, Ethiopia has made a major effort to stimulate and improve efforts under the national salt iodization strategy through regular follow up and monitoring, awareness creation and the rate of salt iodization. In addition, the urban location of the study area could be factors contributing to the higher iodine content observed due to the use of powdered and packed iodized.

In the present study, the proportion of adequately salt in the household was lower as compared to United Nations Children's Fund report from Ruanda (99%), Uganda (99%), Burundi (96%), Burkina Faso (96%) and Kenya (93%) [8]. The possible reason for higher in these countries might be institutionalizing effective iodized salt regulation, updating equipment, and quality-controlled iodization technology at the production, following effective transport channels, correct labeling, packaging, and storage may be explanations for high iodine levels, resulting in adequate amounts of iodine in salt at the households.

Multivariate analysis revealed that the odds of availability of adequately iodized salt was higher among households that had medium and highest wealth status compared to households with poor wealth status. This finding was supported by another study report [18]. This may be due to economically better sections of the household are buying better quality salt in the form of powdered and packed salt. Respondents aged 30-44 years-old 1.99 folds more likely had adequately iodized salt at the household compared with counterparts. This age (30-44 years) might imply the most reproductive age and respondents may have a child and allow households to offer better nutrition for the appropriate child growth and development, which is a tremendous state of affairs that may well contribute to adequate availability of iodized salt.

The present study discovered that secondary school and higher education was associated with increased availability of adequately iodized salt in the household compared to counterparts. Studies conducted in Gondar Town had the same result [14]. This may be because better education may influence good practice; through better awareness about iodized salt.

Respondents having good knowledge about iodized salt and IDD were significantly associated with the availability of adequately iodized salt in the household. The finding was congruent with studies conducted in Gondar [14], Laelay Maychew District [17], and Burie and Wonberma District [20]. The increase in the knowledge levels suggests that, if awareness creation, educational activities, availability, and accessibility of iodized salt are sustained, it is likely that households would become aware of iodized salt and its importance of consuming iodized salt to human health and wellbeing.

Storage of iodized salt in dry places and covering the salt containers were associated with the availability of adequately iodized salt. The findings in Gondar [14], Laelay Michew [17], Kenya [11], and India [9] had the same results. This result verifies that temperature, moisture as well as uncovered storage will affect the viability of the iodine. Because if salt is stored in hummed condition, it attracts moisture and becomes wet, carrying the iodated to the bottom of the container, at hot conditions, salt can release surface moisture, and this may result in iodine loss under its volatility if the container is opened.

## Conclusion

The availability of adequately iodized salt in the household is still low. Further institutionalizing iodized salt regulation and awareness creation will need.

### What is known about this topic

- Groups at the highest risk to suffer from iodine deficiency are lactating women, pregnant women, and children younger than three years;
- The prevalence of goiter is well known;
- A few studies in Ethiopia were conducted to determine the amount of iodine content in the salt at the household level.

### What this study adds

- The availability of adequate iodized salt in the household is still low;
- Educated households were having the availability of iodized salt;
- Covering and storing in a good place were predictors of the availability of adequately iodized salt.
